# Friends and Foes: Bacteria of the Hydroponic Plant Microbiome

**DOI:** 10.3390/plants13213069

**Published:** 2024-10-31

**Authors:** Brianna O. Thomas, Shelby L. Lechner, Hannah C. Ross, Benjamin R. Joris, Bernard R. Glick, Ashley A. Stegelmeier

**Affiliations:** 1 Department of Biology, University of Waterloo, 200 University Avenue West, Waterloo, ON N2L 3G1, Canadaglick@uwaterloo.ca (B.R.G.); 2 Ceragen Inc., 151 Charles St W, Suite 199, Kitchener, ON N2G 1H6, Canadaben@ceragengrow.com (B.R.J.)

**Keywords:** hydroponics, plant pathogens, plant stress, plant growth-promoting bacteria, food production, greenhouses

## Abstract

Hydroponic greenhouses and vertical farms provide an alternative crop production strategy in regions that experience low temperatures, suboptimal sunlight, or inadequate soil quality. However, hydroponic systems are soilless and, therefore, have vastly different bacterial microbiota than plants grown in soil. This review highlights some of the most prevalent plant growth-promoting bacteria (PGPB) and destructive phytopathogenic bacteria that dominate hydroponic systems. A complete understanding of which bacteria increase hydroponic crop yields and ways to mitigate crop loss from disease are critical to advancing microbiome research. The section focussing on plant growth-promoting bacteria highlights putative biological pathways for growth promotion and evidence of increased crop productivity in hydroponic systems by these organisms. Seven genera are examined in detail, including *Pseudomonas*, *Bacillus*, *Azospirillum*, *Azotobacter*, *Rhizobium*, *Paenibacillus*, and *Paraburkholderia*. In contrast, the review of hydroponic phytopathogens explores the mechanisms of disease, studies of disease incidence in greenhouse crops, and disease control strategies. Economically relevant diseases caused by *Xanthomonas*, *Erwinia*, *Agrobacterium*, *Ralstonia*, *Clavibacter*, *Pectobacterium*, and *Pseudomonas* are discussed. The conditions that make *Pseudomonas* both a friend and a foe, depending on the species, environment, and gene expression, provide insights into the complexity of plant–bacterial interactions. By amalgamating information on both beneficial and pathogenic bacteria in hydroponics, researchers and greenhouse growers can be better informed on how bacteria impact modern crop production systems.

## 1. Introduction

The emergence of agricultural practices ~10,000 years ago [1] enabled human populations to expand and form modern civilization [2]. Traditional open-field agriculture remains the most common farming method, with 95% of food grown in soil [3]. However, as a consequence of limited farmland and changes to growing conditions due to climate change, growing crops in greenhouses has emerged as a valuable alternative strategy. The hallmark of greenhouse farming is that crops are grown inside protected structures that mitigate the risks of harsh weather and pests [4]. Collectively, open-field farming and soil-grown greenhouse farming are referred to as conventional agriculture [5]. Mainstream current agricultural methods can cause extensive damage to the finite natural resources needed to sustain current and future food production [6]. For example, tillage, excessive irrigation, monocropping, and chemical applications cause the deterioration of topsoil and soil and water pollution [6,7]. Best practices involving resource optimization and land management are needed to mitigate damage to arable land. Conventional agriculture occupies a substantial amount of land, currently taking up 38% of global land space, and is expected to increase with food demands [8]. To access more fertile land, deforestation and destruction of natural areas will continue. This is a major cause of biodiversity and biomass loss, reducing carbon sinks and, by extension, increasing greenhouse gas emissions [7]. Traditional agricultural practices in soil, such as fertilizer use and over-watering, also release nitrous oxide and methane directly from the soil itself [7]. Decades of unsustainable farming practices have created a feedback loop whereby our actions intensify the effects of climate change, which in turn inhibits our ability to reliably achieve food production targets and manage natural resources [7]. With these obstacles facing conventional agriculture, we need to implement a sustainable solution to ensure we can produce the required amount of food to support a predicted global population of 9.7 billion people by 2050 [9].

A promising alternative to conventional agriculture is hydroponic farming, which utilizes indoor soilless systems to grow plants in a nutrient solution [4]. Crops that are currently grown in hydroponic systems include tomatoes, cucumbers, peppers, leafy greens, and strawberries [10]. The hydroponic sector has experienced significant market growth and is currently worth USD 5.06 billion worldwide annually, with an expected increase to USD 7.36 billion by 2029 via a predicted 7.8% compound annual growth rate [11].

The majority of hydroponic systems are situated within commercial greenhouses, where both natural sunlight and artificial lights are used to support year-round growth [12]. Some hydroponics are utilized in vertical farms, where the systems are stacked to increase plant density per square meter. Vertical farms require extensive artificial lighting to ensure that all layers receive adequate quantities of light. Vertical farms are often not in greenhouses and may receive no natural sunlight; shipping containers and warehouses are commonly used for structures. Both greenhouses and vertical farms are controlled environment agricultural systems, as all aspects of the growth environment are monitored and controlled, including humidity, daily light integrals, introduction of beneficial insects, and airflow.

This rise in the popularity of hydroponics has had a major impact on the agricultural sector, and studies demonstrate hydroponics to be significantly more sustainable compared to conventional agriculture [13]. Hydroponics use up to 90% less water than conventional agriculture [14] by recycling irrigation water and utilizing treated or partially treated wastewater to grow crops [15]. Hydroponic systems can reduce land usage by building on land that is not suitable for traditional agriculture, including areas where the soil is inaccessible, contaminated, experiencing harsh weather conditions, and in urban centers [16]. Producing fresh food in urban areas increases local and nutritious food consumption [1] and significantly decreases the greenhouse gas emissions by food transportation [17]. Reducing water and land usage does not have a negative impact on crop yields. In fact, on average, there is an increase in yield due to the optimization of growth conditions for each crop [18]. Optimizing the number of nutrients added results in a significant reduction in fertilizer usage [5] and, therefore, fertilizer runoff, reducing eutrophication [19]. With hydroponic systems, the risk of soil-borne diseases is also reduced; pesticides can be applied in lower quantities compared to conventional farming [20]. Weeds are also typically a non-issue in hydroponics, reducing herbicide usage and labor [10].

Although the risk of soil-borne disease is lower in soilless systems, plant pathogens can still cause extensive economic damage. Diseases can spread quickly because they are competing for resources with fewer microorganisms, and the shared water source allows for rapid travel through entire systems [21]. Contamination can occur from the water source, employees, insects, air intake, or poor sanitation and sterilization practices [22]. It is difficult to completely eliminate all contamination sources when operating a multi-acre facility. With these challenges in mind, growers develop extensive integrated pest management strategies ahead of a growing season to operate successful hydroponic farms and mitigate sources of contamination.

One aspect of hydroponics that has yet to be fully embraced is the utilization of microbes in hydroponic systems. Plant-microbe interactions are incredibly complex, but understanding them is important to effectively utilize them to manage diseases and maximize yields [23]. Bacteria can be found on every part of a plant, but especially in the rhizosphere, the narrow region located on and around the plant roots [24]. Their relationship with the host plant can be described as symbiotic, pathogenic, or commensal and can influence plant health, flavour [25], stress resistance, and, ultimately, crop yield [26]. In soil, it is known that plant microbiomes can vary depending on factors such as environment, time of year, plant type, stage of growth, soil type, and agronomic practices [23,27,28]. However, plants are also able to directly influence their own microbiome via root exudates [23]. Exudates are fluids that contain both microbial nutrients and signals; their composition varies extensively between crop types and each life stage of a plant’s development. Based on various stimuli such as pests, pathogens, or abiotic factors, plants can secrete different exudates that can attract or repel specific bacteria [29]. Amalgamating current research on the relationship between hydroponic crops and their microbiome is valuable to progress studies in the field and provides growers with the knowledge they need to optimize crop management.

From an agricultural perspective, hydroponic systems are a very different environment from soil, and this extends to the microbiome of the plants [23]. The same microbes that can be found in a plant’s soil microbiome may not be able to access or thrive in a hydroponic environment. Thus, the microbial diversity in a hydroponic system will be vastly depleted compared to the same crop under field conditions. Introducing specific communities of microbes to hydroponic systems may be effective not only to make up for the loss of microbial diversity but also to allow for the design of the microbiome to maximize yield. Increasing our knowledge of plant–bacterial interactions in hydroponics will allow us to utilize the vast capabilities of plant growth-promoting bacteria [27] to increase and improve worldwide food production. In this review, we provide an overview of bacteria that are commercially relevant for hydroponics by discussing seven beneficial and seven pathogenic genera. We also detail different PGPB and pathogenic bacterial pathways, the most current information available on treatments and prevention, and provide commentary on future directions in the field.

## 2. Plant Growth-Promoting Bacteria in Hydroponics

**Figure 1:**
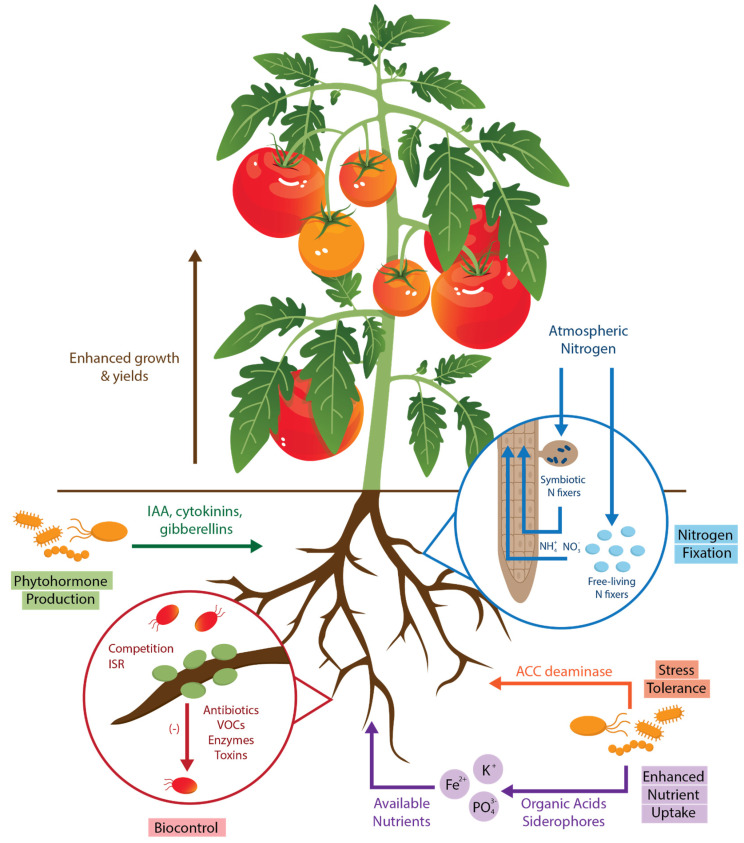
Summary of common plant-growth promotion mechanisms exhibited by PGPB. ACC: 1-aminocyclopropane-1-carboxylate; Fe: iron; IAA: Indole-3-acetic acid; ISR: induced systemic resistance; K: potassium; N: nitrogen; NH4: ammonium; NO3: nitrate; PO4: phosphate; VOCs: volatile organic compounds.

Plant growth-promoting bacteria possess many mechanisms to augment plant growth (Figure 1), either by synthesizing compounds for the plant, facilitating nutrient uptake, or mitigating biotic and abiotic stresses. Some microbes can regulate concentrations of phytohormones, such as gibberellin, auxin, cytokinin, and ethylene in plants [23]. For example, ethylene is associated with decreased plant growth and is triggered by environmental stressors on the plant. The molecule 1-aminocyclopropane-1-carboxylate (ACC) is the immediate precursor of ethylene in higher plants. One known strategy used by some microbes to lower plant ethylene levels is the synthesis of an enzyme called ACC deaminase, which cleaves ACC molecules, leading to a reduction in the ethylene level; therefore, the plant’s growth is not inhibited by these stress events [23]. Another common phytohormone strategy utilizes the auxin molecule indole-3-acetic acid (IAA). IAA influences plant growth, photosynthesis, and many metabolic processes [30]. IAA is also capable of manipulating a plant’s root exudates, thereby contributing to the makeup of the rhizospheric microbiome [23]. Many PGPB are known to produce IAA and can, therefore, heavily influence the plant throughout its entire life cycle [31]. Siderophores are secreted metal chelators produced by a subset of microbes associated with plant growth promotion [23]. Siderophore molecules are able to solubilize iron that is not readily bioavailable in the soil, making it available for uptake by plants while at the same time restricting iron availability to pathogens [23]. In addition to iron, microbes can provide plants with other essential nutrients. Certain bacteria, such as rhizobia, are able to produce ammonia from gaseous nitrogen [23], while others secrete organic acids that solubilize potassium and organic phosphates, making them bioavailable to plants [23]. Additional strategies microbes use to benefit plants include secreting antibiotics or volatile organic compounds (VOCs) that disrupt fungal or bacterial plant pathogens and by out-competing disease-causing organisms [23]. The following section highlights seven key plant growth-promoting genera and summarizes the mechanisms of plant growth stimulation observed in each group with an emphasis on the action of these PGPB in a hydroponic environment (Table 1).

### 2.1. Pseudomonas (Plant-Growth Promoting)

The genus *Pseudomonas* contains both pathogenic and growth-promoting plant-associated strains [65]. Despite their contrasting impacts on plants, these Gram-negative rods often have a striking number of similarities, possessing similar colonization mechanisms and occupying the same niche [66]. Plant growth--promoting strains are commonly found in the species *P. fluorescens*, *P. putida*, *P. protogens*, *P. migulae,* and *P. chlororaphis*, though biocontrol agents can be found in a much broader range of taxa.

There is substantial evidence that *Pseudomonas* species are growth--promoting agents in hydroponics, possessing the ability to improve plant growth even under optimal growing conditions [34]. For example, inoculation of hydroponic lettuce grown in a nutrient film technique (NFT) system with *P. lundensis* and *P. migulae* significantly enhanced several agronomically important parameters, including shoot fresh weight, number of leaves, leaf area, and fresh and dry root weight [34]. Due to the increased accumulation of IAA in the leaves and roots of inoculated plants, this growth promotion was primarily attributed to the enhancement of plant IAA production by both *Pseudomonas* species [34]. *Pseudomonas* sp. LSW25R demonstrated plant growth promotion in hydroponically grown Momotero tomatoes, eggplants, and hot peppers [67]. However, optimal growth was observed at different inoculum concentrations for each crop. Tomatoes displayed the greatest height and fresh weight when treated with 10^8^ CFU ml^−1^ of *Pseudomonas* sp. LSW25R, whereas eggplant and hot pepper seedlings showed maximum growth at the lower dose of 10^4^ CFU ml^−1^. This suggests variation in the optimal population density of bacterial inoculum between plant species in a hydroponic environment. In addition to IAA production, there are several mechanisms thought to contribute to the enhancement of plant growth by *Pseudomonas,* including siderophore production, nitrogen fixation, and ACC deaminase activity [32]. A study performed on Green Oakleaf, Rex, Red Oakleaf, Red Sweet Crisp, Nancy, and Red Rosie lettuce cultivars grown in an NFT system showed that *P. psychrotolerans* IALR632 significantly increased the shoot fresh weight of all cultivars but Red Rosie, with a maximum increase of 55.3% being observed in Green Oakleaf [68]. Green Oakleaf plants inoculated during germination also displayed a 164% enhancement of lateral root development compared to uninoculated controls. In vitro tests revealed that the *P. psychrotolerans* IALR632 possessed a multitude of growth-promoting traits, including auxin production, ACC deaminase activity, nitrogen fixation, and phosphate solubilization [32], which were thought to contribute to the observed growth promotion of the hydroponic crops [68].

Along with directly promoting plant growth, some *Pseudomonas* spp. have been shown to be effective biocontrol agents in hydroponic systems, equipped with multiple mechanisms for disease reduction. This ability has been exploited in several products that are commercially available in North America and Europe [69]. Biocontrol mechanisms employed by these *Pseudomonas* spp. include antibiotic production, competition for nutrients and niches, and the production of lipopeptides, which may act directly against phytopathogens or provoke lipopeptide-mediated induced systemic resistance [35]. Certain pseudomonads, such as *P. putida* and *P. fluorescens*, use type VI secretion systems for the biocontrol of plant pathogenic bacteria [36]. This system has also been suggested to have roles in limiting the growth of fungal phytopathogens [37], which represent a number of the most common plant pathogens found in hydroponic systems. Type VI secretion systems exhibit antimicrobial effects through the injection of toxins into target cells [36]. These systems are also important for the persistence and establishment of *Pseudomonas*, allowing them to outcompete other microbes in the rhizosphere [36]. There are strain-specific differences in biocontrol capabilities, as has been observed in *P. protegens* Pf4 and Pf11 strains isolated from the roots of hydroponically grown lamb’s lettuce [70]. Both strains exhibited broad-range fungal inhibition, although the inhibitory effect of Pf4 was much stronger [70]. This was attributed to Pf4-containing gene clusters for the synthesis of the secondary metabolites pyoluteorin, pyrrolnitrin, and rhizoxin [70]. Pyoluteorin is an antibiotic that is effective against bacteria, oomycetes, and fungi. Of note, it is effective against the devastating pathogen *Pythium*. Pyrrolnitrin is an antifungal that inhibits the electron transport system. Rhizoxin is an antibiotic that also demonstrates anti-tumour potential due to targeting the microtubules during mitosis to inhibit cell division. It should also be noted that in vitro biocontrol tests may not be consistent in providing an accurate assessment of the biocontrol abilities actually exhibited in hydroponic systems. This is in part due to the dilutive nature of such systems potentially reducing the impact of secondary metabolites [36], but may also be due to bacterial strains exhibiting differential modes of biocontrol in vitro as opposed to in planta [37]. In a study examining the biocontrol abilities of 49 *Pseudomonas* strains against the bacterial pathogen *Agrobacterium rhizogenes*, of the 13 strains that exhibited in vitro antagonism, only three were effective biocontrol agents in planta [37]. Additionally, six strains that did not show significant in vitro growth inhibition of *A. rhizogenes* displayed a reduction in disease incidence greater than 50% when applied to tomato plants [37]. While the in vitro tests were effective in identifying the most active biocontrol strains, these findings suggest that the use of a single in vitro test does not provide a comprehensive view of the biocontrol activity of a given strain.

The above biocontrol capabilities of *Pseudomonas* species have been shown to reduce the incidence and severity of a number of diseases in a variety of hydroponic crops. In hydroponic cherry tomatoes (cv. Money Maker), *P. protegens* and *P. brassicacearum* were able to reduce the incidence of hairy root disease (HRD) caused by *Agrobacterium rhizogenes* by 80%, while *P. clororaphis* reduced disease incidence by 50% [37]. Genomic analysis revealed the presence of genes involved in the type VI secretion system in each strain, suggesting that this mechanism played a role in the observed *A. rhizogenes* inhibition [37]. Additionally, *Pseudomonas* species reduced the severity of root rot caused by *Pythium ultimum* in hydroponic tomatoes (*Lycopersicon esculentum* Mill. ‘Trust F1′) by up to 22.8% and decreased the rate of infection four months after pathogen inoculation by up to 77.8% [38]. Inoculation with *P. fluorescens*, *P. marginalis*, *P. putida*, and *P. syringae* also resulted in significantly greater individual fruit mass, marketable and total yields, effectively minimizing the economic loss associated with root rot in hydroponic systems [37] In hydroponic zucchini grown in a fully automated closed soilless system, application of *Pseudomonas* PB26 reduced the severity of *Phytophthora* crown rot by 30%, while a mixture of three *Pseudomonas* strains (FC7B, FC8B, FC9B) isolated from a soilless rockwool medium reduced disease severity by 42% [71].

### 2.2. Bacillus

*Bacillus* species are rod-shaped, Gram-positive bacteria characterized by their endospore-forming capabilities and aerobic growth [72]. With such broad defining characteristics, extreme phenotypic diversity is observed within the >200 known species of the genus. *Bacilli* are ubiquitous in nature and display a wide range of physiological capabilities [72]. This diversity lends itself well to a variety of commercial applications, with various members of the genus being used in the food, brewing, and paper industries [72]. One such application is the use of *Bacillus* spp. as a biofertilizer and biocontrol agent for hydroponically grown crops. Several species have been noted for their plant growth-promoting capabilities, including *B. subtilis*, *B. velezensis*, *B. amyloliquefaciens*, *B. cereus*, and *B. pumilus*. Along with exhibiting traditional plant growth-promoting traits, the endospore-forming ability of *Bacillus* is beneficial for their use in commercial biofertilizers [73]. Endospores offer enhanced strain survival in adverse conditions, thus extending the product shelf life and permitting the formulation of bacterial consortiums with an antagonistic relationship [73].

*Bacillus* species employ many direct mechanisms of plant growth promotion that may be observed in the hydroponic environment. This includes the production of ACC deaminase, with several halotolerant *Bacillus* strains being shown to stimulate growth in saline-stressed rice [39], cucumber [74], tomato [75], beans [76], and wheat [77]. Phytohormone synthesis also plays a crucial role in *Bacillus*-mediated plant growth promotion. Numerous *Bacillus* species are known to produce IAA [43,44], cytokinins [78], and gibberellins [79], all of which result in enhanced plant growth and increased yields but employ different modes of action [42]. Cytokinins extend the leaf photosynthetic period of plants by promoting cell division [80], whereas gibberellins promote shoot elongation and seed germination [81]. Siderophore production, as well as phosphate and zinc solubilization by *Bacillus* species, has been observed in vitro and thus is another potential mechanism for growth promotion via enhancing plant nutrition [43]. Several species of *Bacillus* have also exhibited nitrogen-fixing capabilities. In a study examining 18 *Bacilli* isolated from a tropical region, all but three species studied were shown to possess the *nifH* gene, including the plant growth-promoting species *B. subtilis* and *B. cereus* [82]. *B. megaterium* and *B. mycoides* have also been demonstrated to possess *nifH* genes and fix nitrogen in the sugarcane rhizosphere [83]. A ^15^N_2_ isotope dilution experiment showed that inoculated plants have significantly higher N content, while qRT-PCR analysis revealed that the highest levels of *nifH* expression occurred 60 days post-inoculation [83]. Though nitrogen fixation by *Bacilli* has yet to be thoroughly examined in a hydroponic environment, inoculation of hydroponic lettuce with *B. subtilis* at densities of 7.8 × 10^3^ and 15.6 × 10^3^ CFU mL^−1^ has been demonstrated to increase plant N accumulation, which may be attributed to biological nitrogen fixation (BNF) [84].

While the PGP (plant growth promoting) capabilities of *Bacillus* have primarily been determined in vitro or demonstrated in soil environments, several studies have highlighted the potential of *Bacillus* inoculation in enhancing the growth of hydroponic crops. Combined inoculation of *B. subtilis* with *Arthrobacter pascens* at a concentration of 5 × 10^5^ CFU mL^−1^ markedly increased the yield of lettuce and celery grown in a vertical hydroponic system, resulting in a 1.98- and 1.3-fold increase in above-ground fresh weight after 30 and 45 days, respectively [85]. Inoculation also increased plant nutrition, exhibiting increased concentrations of protein, vitamin C, and phenol in both vegetables [85]. Both lettuce and celery experienced enhanced root nutrient uptake and leaf photosynthesis as a result of microbial inoculation, as evidenced by increased root dehydrogenase activity, stomatal conductance, and net photosynthetic rate [85]. Inoculation of iceberg lettuce grown in a hydroponic NFT system with *B. subtilis* yielded similar results, with a 25% increase in leaf yield, a 20% increase in leaf number, and a 22% increase in shoot fresh matter being observed in plants inoculated with 15.6 × 10^3^ CFU mL^−1^ *B. subtilis* [84]. Mitigating the deleterious effects of abiotic stresses on plants serves as another important growth-promoting trait that the *Bacillus* genus employs in hydroponic systems. In hydroponic cucumbers (*Cucumis sativus* L. cv. Jinchun No. 2) under salinity stress caused by four times strength nutrient conditions, inoculation of the growth substrate with *B. subtilis* K424 improved growth 1.08–1.14 times compared to uninoculated plants [86]. *B. subtilis* inoculation alleviates the toxic effects caused by high nutrient concentrations, enhancing the photosynthetic rate by ~18% and stomatal conductance by ~29% [86]. Interestingly, the *B. subtilis* inoculant may influence the microbial community structure in the cucumber hydroponic root microbiome [86]. While 16S sequencing showed that all plants grown under high salinity conditions exhibited a lower Shannon index (a measure of microbial diversity) for bacteria than non-stressed controls, inoculated plants had an increased relative abundance of plant growth-promoting genera such as *Rhodobacter*, *Bacillus*, and *Pseudomonas* [86]. However, it should be noted that the heightened abundance of *Bacillus* was likely due to the inoculation itself persisting throughout the 60-day growth period and not the recruitment of additional *Bacillus* as a result of inoculation. Regardless, inoculated plants under salinity stress displayed a higher Shannon index for bacterial diversity compared to uninoculated salt-stressed controls, indicating that *B. subtilis* partially restored the diversity loss associated with high salinity conditions [86].

*Bacillus* indirectly promotes plant growth in hydroponic systems by protecting plants from infection by phytopathogens. While siderophore and antibiotic production contribute to this function, one of the primary means of biocontrol employed by *Bacilli* is the production of the lipopeptides of the surfactin, fengycin, and iturin families [87]. These secondary metabolites bind to the cell membrane, inducing transient membrane disruption or local bilayer disorder, thus causing permeability changes and, ultimately, cell death [88]. As such, these compounds have antimicrobial and antifungal properties that equip some *Bacillu*s species with biocontrol capabilities [89]. Fengycin and surfactin can also grant pathogen resistance by eliciting induced systemic resistance in a variety of plants, thereby providing protection against a broad range of phytopathogens [90]. Such mechanisms have been shown to be effective against a number of agriculturally significant phytopathogens found in hydroponic environments. Various strains of *B. velezensis* have exhibited both antibacterial and antifungal properties, displaying effective control against several diseases that may cause substantial economic losses in hydroponic tomatoes, including bacterial canker caused by *Clavibacter michiganensis* [91], *Botrytis cinerea* [92], as well as Fusarium crown and root rot [44]. *B. velezensis* exhibited biocontrol of the fecal coliform *Escherichia coli* in hydroponic lettuce due to the production of surfactin and fengycin, further demonstrating its potential as a hydroponic biocontrol agent [89]. *B. subtilis* strain QST 713 is also an effective biocontrol agent in hydroponics, reducing the incidence of powdery mildew, gummy stem blight, and Fusarium root and stem rot in hydroponically grown cucumbers [45]. Additional mechanisms of biocontrol exhibited by *Bacillus* species are the production of VOCs, namely alcohols, aldehydes, ketones, and benzothiazoles, which can activate ISR and/or directly inhibit pathogens [93]. *Bacillus* species are also capable of indirect pathogen control via niche competition [45], which has been exhibited by *B. subtilis* SQR 9 in controlling Fusarium wilt in cucumbers [94]. In addition to the production of lipopeptides, the incidence of Fusarium rot was reduced by 49–61% due to the high densities of this bacterium colonizing the cucumber roots, thereby preventing the root colonization of *Fusarium* and providing intense competition for nutrients and niches [94].

### 2.3. Azospirillum

First discovered by Beijerinck in 1925 [95], the *Azospirillum* genus is one of the most extensively studied groups of PGPB [96]. *Azospirillum* were initially defined by their nitrogen-fixing capabilities and phytohormone production, and as such, these processes were long considered to be the main modes by which the genus promoted plant growth [96]. However, further research has elucidated that *Azospirillum* harbors a more diverse set of growth-promoting mechanisms than was initially realized [96]. Some *Azospirillum* strains are capable of ACC deaminase production [46], phosphate solubilization [51], siderophore production [48], enhancing nutrient uptake by plants [52,97], induction of abiotic stress tolerance, and induction of pathogen defense mechanisms [98]. While the vast majority of studies examine *Azospirillum* in a soil environment, the genus has demonstrated potential as a plant growth--promoting agent in hydroponic systems through the same strategies. Due to the wide variety of mechanisms employed by the *Azospirillum* genus, their mode of plant growth promotion is best described by the Multiple Mechanisms Theory [96]. This theory posits that there is no single mode by which *Azospirillum* promotes plant growth; instead, growth promotion occurs due to the additive effects of multiple mechanisms, which may vary based on the plant species [96]. It can be applied to many other PGPB genera, including *Pseudomonas* and *Bacillus,* which possess an arsenal of mechanisms to reduce stress, increase nutrient uptake, and prevent colonization by phytopathogens. Different mechanisms would only function optimally under specific environmental conditions. Thus, diversifying strategies to improve plant growth ensures that the bacteria can assist the plants throughout the varying external conditions that present throughout a growth cycle spanning multiple months.

*Azospirillum* are free-living nitrogen fixers, with *A. palatum* serving as the only member of the genus that has so far not demonstrated the ability to fix nitrogen [99]. While biological nitrogen fixation was first discovered and is among the most commonly cited mechanisms for *Azospirillum* plant growth promotion, it is also the most controversial [96]. Many studies have brought the agronomic significance of this mechanism into question, showing that *Azospirillum* typically causes only around a 5% increase in plant nitrogen content [96]. This has been validated in hydroponic sweet peppers, determining that inoculation with *Azospirillum* and *Pantoea* resulted in minimal transfer of fixed nitrogen to the plants [47]. It should be noted that *Pantoea* does not have an effect on plant N nutrition and was added because increased growth stimulation and nutrient uptake in plants has been observed when *Azospirillum* is co-inoculated with this bacterium [47]. Despite this, nitrogen fixation as a growth-promoting mechanism should not be discarded. Numerous studies have reported significant contributions of nitrogen fixation by *Azospirillum* to total plant N content [100,101], showing increases of up to 39% [102].

*Azospirillum* produces several plant growth regulators, including auxins [96], gibberellins [96], cytokinins [46], ethylene [103], nitric oxide [104], and polyamines [105]. Much of the growth-promoting capabilities of *Azospirillum* have been accredited to IAA production, which may be considered the most important rhizospheric function of the genus [49]. However, there is variation among *Azospirillum* strains and their ability to produce IAA. In a study examining 50 *Azospirillum* isolates obtained from maize soils, 88% exhibited poor IAA production abilities [106]. Several factors also impact IAA production by *Azospirillum*: limited carbon supply triggers the production of the phytohormone, while aerobic conditions dramatically reduce its production [107]. Maximum IAA production can be seen in microaerobic conditions and during the bacterial stationary phase when the greatest microbial biomass is observed [49]. Along with directly promoting plant growth, *Azospirillum* plays an important role in the mitigation of adverse effects due to abiotic stress. While *Azospirillum* is naturally present in the rhizosphere of a wide variety of plants, several studies have demonstrated that the genus can be selectively recruited to plants under stress conditions due to altered root exudate patterns and plant defense mechanisms, resulting in a higher relative abundance [108]. When the nettle Ramie was subjected to biological stress, it contained a higher relative abundance of *A. lipoferum* and *A. brasilense* in their rhizosphere, which was attributed to the regulation of metabolites including orthophosphate, uracil, and Cys-Gly [109].

While the *Azospirillum* genus consists of over 30 species, the first described members, *A. lipoferum* and *A. brasilense*, remain the best characterized and most widely used in commercial products. *Azospirillum* has been used commercially as a biofertilizer for field crops in South America, Mexico, Europe, South Africa, and India [110]. In 2015, there were 104 biological products containing *Azospirillum* on the South American market, all of which were formulated with *A. brasilense* [111]. Limited research is available on the other *Azospirillum* species and their potential as biofertilizers. Applications of *Azospirillum* in hydroponics are limited to growth promotion studies in select crops and have not yet been applied on a large scale or exploited for commercial use. In hydroponically grown strawberries, *A. brasilense* has been found to enhance nutrition [48,97] and potentially confer pathogen resistance [98]. Inoculation with *A. brasilense* significantly increased the concentration of manganese, copper, and zinc in the fruits [97], and siderophore production by *A. brasilense* REC3 contributes to the iron nutrition of the plant [48]. Several studies have also shown *Azospirillum* to be effective in enhancing yields and nutrient acquisition in hydroponic lettuce and arugula. A single foliar application of *A. brasilense* at a dose of 300 mL 250 L^−1^ to hydroponic arugula 10 days after seedling transplantation into an NFT system resulted in heightened concentrations of N, P, K, Ca, Mg, and S in the leaves [112]. Such effects result in biological biofortification and thus provide benefits for human nutrition [113]. Similar results were obtained with hydroponic iceberg lettuce, with a single liquid *Azospirillum* inoculation on the day of transplantation improving the accumulation of N, P, K, S, Ca, Mg, B, Cu, Zn, Fe, and Mn by 39–67% [52]. The optimal dose of inoculant varied for the accumulation of each nutrient, with the highest tested dose (61 mL 100 L^−1^) impairing the plant’s nutritional balance^61^. However, a dose of 44 mL 100 L^−1^ gave rise to the maximum leaf yield while maintaining plant nutrition [52].

### 2.4. Azotobacter

*Azotobacter* is a globally distributed group of non-symbiotic nitrogen-fixing bacteria, typically found in 30–80% of soils sampled worldwide [53]. There are currently nine members of the genus: *A. vinelandii, A. chroococcum, A. paspali, A. tropicalis, A. nigricans, A. salinestris, A. bryophylli, A. beijerinckii,* and *A. armeniacus*. While the genus as a whole is regarded for its plant growth promotion capabilities, *A. chroococcum* is the most commonly isolated and, thus, most widely studied member. The diverse array of mechanisms of plant growth stimulation exhibited by *Azotobacter* and the well-established role of the genus as a PGPB in the soil environment highlight the potential for use as a growth-promoting agent in hydroponics. These mechanisms include biological nitrogen fixation, phytohormone production [54], and phosphate solubilization [54]. Siderophore production also acts as a PGP capability of the genus, with select *Azotobacter* producing the fluorescent-green siderophore azobactin under iron-limited conditions, while *A. vinelandii* has been shown to synthesize the non-fluorescent catchetol-type siderophores zotochelin, protochelin, and aminochelin [53]. Unlike many other groups of PGPB, *Azotobacter* has not been documented to exhibit ACC-deaminase production [54]. One of the most unique features harbored by the *Azotobacter* genus is the ability to form cysts in unfavorable conditions [114]. The structures confer a heightened resistance to drying, UV light, ultrasound, as well as gamma and solar irradiation compared to vegetative cells [114]. Cyst formation occurring within a biofertilizer containing *Sinorhizobium meliloti, A. brasilense*, and *A. lipoferum* extended the viability of the strains and, thus, the shelf life of the product without sacrificing its efficacy [115]. Thus, the capacity to form cysts makes *Azotobacter* species ideal candidates for hydroponic biofertilizer formulations, similar to endospore production in *Bacillus*.

*Azotobacter* species are known for their nitrogen-fixing capabilities and are characterized by their extreme oxygen tolerance when performing nitrogen fixation. There are several proposed mechanisms this trait is thought to arise from, including respiratory protection of nitrogenase [116], physical blockage of oxygen by the formation of alginate capsules on the cell surface [117], and the conformational protection of nitrogenase via association with a FeSII protein [118]. As such, *Azotobacter* species are obligate aerobes that are able to carry out nitrogen fixation under oxygen-rich conditions, a trait that is advantageous for use as a biofertilizer in the aerobic environment of a hydroponics system. Similar to *Azospirillum*, *Azotobacter* species are free-living nitrogen fixers, though their influence on total plant N levels has not been as extensively studied. Co-inoculation of *A. chroococcum* strains AC1 and AC10 reduced required nitrogen fertilization doses in cotton in its flowering stage by up to 50% but only increased plant nitrogen content by a maximum of 4% [54].

The growth-promoting capabilities of *Azotobacter* have primarily been examined in field conditions and in vitro, with limited research available on the use of *Azotobacter* in a hydroponic environment. However, the available studies have shown *Azotobacter* to increase yields in hydroponically grown strawberries and lettuce. Hydroponically grown strawberry plants inoculated with *Azotobacter* and doses of 100 or 150 ppm nitrogen exhibited the greatest yield in relation to both the controls and other inoculated plants [119]. Foliar application of *Azotobacter* on hydroponic lettuce at seven-day intervals for a total of one month was determined to increase the fresh and dry weight of the crop, as well as leaf number and leaf area under three different nitrogen levels (100, 150, and 200 mg L^−1^ in nutrient solution) [64]. Treatment of lettuce plants with *Azotobacter* at 200 mg L^−1^ nitrogen displayed the largest increase in leaf area of 7.8% compared to the uninoculated plants grown under the same added nitrogen level. Conversely, *Azotobacter* inoculation did not enhance agronomic parameters such as weight and fruit number in hydroponic tomatoes (*Lycopersicon esculentum* Mill. Var. Valoasis RZ), but inoculation was correlated with higher quantities of grade A fruit [120].

### 2.5. Rhizobium

*Rhizobium* represents one of 14 genera of symbiotic nitrogen-fixing bacteria that associate with the roots of legumes [121]. In contrast to the previously discussed genera, *Rhizobium* is an endophytic PGPB, inhabiting nodules formed in plant roots [56]. This represents a mutualistic relationship in which *Rhizobium* fixes atmospheric nitrogen and provides ammonia to the plant, receiving fixed carbon compounds in return [122]. The PGP capabilities of *Rhizobium* have been long known, with the first-ever bioinoculant, Nitragin^®^, patented by Nobbe and Hiltner in 1895 [123], containing *Ensifer meliloti* (formerly *Rhizobium meliloti*) as its primary active ingredient [124]. Since then, the genus has acted as a primary constituent of many early biofertilizers and maintains a prominent role in formulations to this day [124]. However, a major limitation of the commercial use of *Rhizobium* is the extreme specificity of the legume-*Rhizobium* symbiosis, especially considering that domesticated crops have been observed to exhibit a higher specificity in this interaction [125]. Even if a nodule is successfully formed, the efficiency of nitrogen fixation may vary vastly between different bacterial and plant partners [125]. This symbiosis also presents potential limitations for the use of *Rhizobium* in hydroponic systems, as the genus can only be used as a growth-promoting agent in leguminous crops, which do not currently comprise a large proportion of the hydroponics market.

Symbiotic nitrogen fixation accounts for an estimated 55–78% of BNF globally, with the exact proportion of symbiotic to free-living BNF varying between biomes [126]. It is an incredibly efficient process, as up to 90% of the nitrogen fixed is utilized by the host plant [127]. As such, symbiotic nitrogen fixation represents the most widely known and perhaps the most significant contribution of *Rhizobium* to plant growth promotion. Along with BNF, *Rhizobium* exhibits many other direct and indirect mechanisms of plant growth promotion. This includes the production of IAA, cytokinins, and gibberellins, as well as the solubilization of organic and inorganic phosphate via 2-ketogluconic acid production [58]. Siderophore production by *Rhizobium* has been shown to enhance plant iron nutrition and inhibit the growth of fungal phytopathogens such as *Fusarium solani, Macrophomina phaseolina,* and *Rhizoctonia solani* [57]. *Rhizobium* also exhibits other mechanisms of biocontrol against fungal pathogens, including nutrient competition, mycoparasitism, and the production of antifungal compounds, including the antibiotics trifolitoxin and rhizobitoxin, as well as mycolytic enzymes and HCN [121].

Despite serving as the most well-characterized nitrogen-fixing bacteria, few studies have been conducted on the impacts of *Rhizobium* inoculation on the growth of plants in hydroponic systems. Current research is limited to the effects of *Rhizobium* on enhancing yields of soybean and common bean in hydroponics. Inoculated hydroponic common bean was grown under a reduced N supply with *Rhizobium sophoriradicis* by submerging seedlings in liquid culture (10^9^ CFU mL^−1^) for ten minutes, followed by watering seedlings with an inoculum containing 10^8^ CFU mL^−1^ at a rate of 10 mL plant^−1^ seven days after sowing. *Rhizobium sophoriradicis* minimized yield loss under a 64% reduced inorganic N supply, resulting in losses of 11.2% compared to the 35% loss observed in the uninoculated control [122]. The combined effects of *Rhizobium* inoculation and sulfur supplementation have also been shown to enhance yields by 30.9% to 119.4% in hydroponic soybean at sulfur concentrations of 0.15, 0.30, and 0.60 mM of sulfur [128]. This result may be attributed to sulfur deficiency reducing root nodulation in soybean [128]. Along with directly promoting plant growth, *Rhizobium* may hold potential as a biocontrol agent in hydroponics. *Rhizobium* sp. strain TBD182, a strain isolated from a hydroponics system, exhibits inhibitory effects against the mycelial growth of *Fusarium oxysporum* [129]. Genomic analysis of this strain provided insight into the potential mechanisms of this fungistatic activity, including the production of fungal cell wall degrading enzymes and the potential role of various secretion systems [129].

### 2.6. Paraburkholderia

The genus *Burkholderia* initially consisted of bacteria pathogenic to plants, animals, and humans, as well as plant growth--promoting bacteria [130]. As a result of this differing pathogenicity and multiple phylogenetic analyses, the genus was divided into six groups: *Paraburkholderia*, *Burkholderia*, *Trinickia*, *Robbsia*, *Mycetohabitans*, and *Cabelleronia* [63]. *Burkholderia* now consists of the pathogens and opportunistic pathogens of the original genus, whereas *Paraburkholderia (*a monophyletic clade within the *Burkholderiaceae* family with conserved signature indels unique to *Paraburkholderia* species) is primarily composed of plant growth-promoting species [63]. Prior to the division of the genus, *Burkholderia* was commercialized, serving as the primary active ingredient of several products used for pest control and plant growth promotion, along with other industry applications [131]. However, the discovery of the pathogenicity of many members of the genus ultimately resulted in the withdrawal of such formulations [131]. The reclassification of *Paraburkholderia* holds promise for these bacteria to be used commercially once again with potential applications in hydroponic systems, provided that the biosafety potential of the group is more firmly established [131].

*Paraburkholderia* exhibits a wide range of plant growth-promoting strategies; however, no single mechanism has been observed in all members of the genus [63]. Biological nitrogen fixation is a common growth promotion characteristic within *Paraburkholderia*, with about half of the known nitrogen-fixing species in the genus also capable of nodule formation [63]. In addition to nitrogen fixation, *Paraburkolderia* exhibits many other mechanisms of growth promotion. ACC deaminase production by both rhizosphere and phyllosphere colonizing *Paraburkholderia* has been demonstrated to be effective in lowering ethylene levels and thus promoting growth [62]. IAA production has also been observed in many members of the genus, including *P. kururiensis*, *P. phytofirmans*, *P. unamae*, and *P. tropica* [64]. Other mechanisms of plant growth promotion exhibited by *Paraburkholderia* include siderophore production, phosphate solubilization, and antifungal activity [63].

Limited studies have been conducted on the plant growth-promoting effects of *Paraburkholderia* in hydroponic crops; however, strains of *Paraburkholderia* sp. and *Burkholderia contaminans* have been demonstrated to enhance hydroponic lettuce growth under heat stress [132]. This study utilized the butterhead lettuce varieties ‘Buttercrunch’ and ‘Rex’, which were inoculated with 1.0 mL of liquid culture one week before transplantation into a hydroponic system. Plants were exposed to heat stress throughout the summer season, where the mean daytime temperature was 7 °C above the upper threshold of their optimal range (12–18 °C). After 15 days, Rex plants inoculated with *Paraburkholderia* strain IALR387 and *Burkholderia contaminans* strain IALR1819 displayed a 43% and 60% increase in shoot fresh weight, respectively. These results can primarily be attributed to the high ACC deaminase levels exhibited by the tested strains, which were effective in reducing ethylene levels produced by the lettuce plants under heat-stress conditions [132]. Other mechanisms of growth promotion exhibited by the tested endophytes include IAA production, phosphorus solubilization, siderophore production, and nitrogen fixation [132].

### 2.7. Paenibacillus

Formerly known as “group three” of the genus *Bacillus*, several *Paenibacillus* species have displayed promise as plant growth-promoting agents in hydroponic systems [133]. The genus is primarily regarded for its biocontrol capabilities but also harbors other PGP traits. Of the over 174 members of the genus [133], over 20 *Paenibacillus* species are capable of nitrogen fixation [59]. However, nitrogen-fixing activity varies greatly from species to species. Acetylene reduction assays demonstrated that *P. zanthoxyli* DSM 18202 had 140 times greater activity than *P. peoriae* DSM 8320 [134]. Additionally, *Paenibacillus* performs phosphate solubilization via gluconic acid production. Genomic analysis of 35 *Paenibacillus* genomes demonstrated that all but two harbored the phosphate solubilization genes encoding glucose-1-dehydrogenase and gluconic acid dehydrogenase [61]. IAA production serves as another important mechanism of plant growth promotion by *Paenibacillus*, with genomic analysis suggesting that the genus primarily uses the indole-3-pyruvic acid pathway [61]. *Paenibacillus* also contributes to plant iron nutrition and exhibits pathogen antagonism through the production of siderophores [60].

Hydroponic studies of *Paenibacillus* are primarily focused on the biocontrol abilities of the genus. *Paenibacillus* species have been shown to produce a large variety of peptide antibiotics, including polymyxin, tridecaptin, paenilan, and fusaricidin [135]. Other mechanisms of biocontrol exhibited by *Paenibacillus* include the induction of systemic resistance in host plants and niche competition [136]. The microbial community of healthy hydroponic tomatoes (*Solanum lycopersicum* L. with Maxifort and DRO141TX rootstocks) has been contrasted with those inflicted by hairy root disease (HRD), with particular attention to the causative agent of HRD, *Agrobacterium*, and *Paenibacillus* populations [137]. It was determined that compared to greenhouses infected with HRD, *Paenibacillus* was significantly associated with non-infected greenhouses, suggesting the ability of endogenous *Paenibacillus* to prevent *Agrobacterium* infection in hydroponic tomatoes [137]. Further, while *Paenibacillus* is naturally present in the root microbiome of hydroponic tomatoes, additional inoculation was observed to reduce the incidence of HRD in two-headed beef tomatoes [138]. Following transplantation into rockwool cubes, 50 mL of inoculum at a density of 10^5^ to 10^7^ was applied to the plants for 10 consecutive days, after which *Paenibacillus* and *Agrobacterium* were applied once a week for four weeks. Co-inoculation of *P. xylanexedens* strains ST15.15/027 and AD117 at densities of 10^5^ to 10^7^ were observed to be equally effective for biocontrol in the hydroponic system, reducing HRD infestation rate by 23–36% [138]. *Paenibacillus* has also demonstrated potential as a biocontrol agent in other hydroponic crops. Inoculation with *P. polymyxa* SQR-21 at a concentration of 1 × 10^6^ CFU mL^−1^ exhibited biocontrol effects against *Fusarium* wilt disease in hydroponic watermelon by eliciting differential expression of 119 proteins involved in growth, defense, signal transduction, transportation, metabolic processes, and stress response [139].

## 3. Phytopathogenic Bacteria in Hydroponics

As growers are acutely aware, not all microbes enhance plant growth, and some microbial infections can cause devastating crop losses. Biotrophic pathogens take up nutrients from living cells and thus must keep their host alive at least temporarily, while necrotrophic pathogens absorb nutrients from dead cells, quickly causing necrosis of the plant once infected [140]. Many pathogens use both of these strategies at varying stages in their life cycle. Some pathogens harm plants by secreting enzymes or phytotoxins that break down cell walls or other vital plant cell components [141]. The proliferation and multiplication of bacterial cells can cause blockages in plant vessels, decreasing the plant’s ability to uptake water or nutrients and rapidly causing severe wilting and death [142]. Some pathogenic microbes have evolved to manipulate a plant’s immune responses. For example, stomata and plasmodesmata can be overridden by microbial biochemical mechanisms to allow pathogen entry and colonization [143,144,145,146], and plant communication pathways such as calcium signaling can be targeted and controlled [147]. There are a variety of mechanisms used by plant pathogens when infecting their host, and crop outcomes depend on the location and timing of the infection [148]. Furthermore, bacteria can be opportunistic phytopathogens. These organisms survive in hydroponics systems and typically do not cause disease. However, bacteria may infect the plant if the opportunity arises, such as mechanical damage to plants, an alteration in the microbiome, a change in humidity/temperature, or a reduction in a plant’s induced systemic resistance (ISR).

There are many bacteria that cause disease in a wide range of economically important hydroponic crops (Table 2) and display great host specificity. A bacterium that causes disease in one plant species may have no impact on a different crop [149]. In the following section, seven genera of bacteria that are economically relevant in hydroponic crops have been outlined with descriptions of their pathogenicity and disease management strategies.

### 3.1. Xanthomonas

*Xanthomonas*, meaning yellow monad in Latin [186], is a large and well-documented genus [150]. The Gram-negative bacterium is known for its unique color, which results from the production of xanthomonadin [186]. This genus possesses three of the top 10 most phytopathogenic bacteria [187], including *X. campestris* infection of hydroponic brassicas. The genus *Xanthomonas* consists of over 35 distinct species and has been reported to cause disease in over 400 plant species, including hydroponic tomatoes and peppers [186]. The optimal temperature for growth is between 25 and 30 °C; high humidity contributes to faster spread of disease symptoms [150]. Indeed, relative humidity >90% enables *Xanthomonas* to reach infection sites and multiply more rapidly. Free water from fog or irrigation systems also contributes to disease progression. Twenty-four hours of consistent moisture on plant leaves doubled the number of lesions caused by *Xanthomonas citri* on Tahiti lime crops [188]. These characteristics contribute significantly to the geographical distribution of *Xanthomonas* and its ability to cause pervasive disease in greenhouse conditions.

The plant pathogen *Xanthomonas* is responsible for many different diseases, both pre- and post-harvest. Depending on the type of plant and strain, *Xanthomonas* can cause blight, spots, cankers, and rot [150]. *Xanthomonas* has a wide range of virulence factors and secretion systems that contribute to host specificity. The bacterium is predominately seed-borne and has the ability to colonize both the plant vascular system and its mesophyll tissue [186]. Transmission occurs most often through contaminated seeds but can also spread via weeds, environmental factors including airflow and irrigation, or directly from infected plants [150]. Entering through stomata or surface wounds, the bacterium primarily uses the type II and III secretion systems to deliver cell-wall degrading enzymes that facilitate pathogenicity. Phylogenomic analysis reveals the use of a type VI secretion system in some species [186]. Tomatoes, strawberries, lettuce, peppers, and watercress [150] are the most common hydroponically grown plants affected by the bacterium [153,189]. While different hosts exhibit a slight variation in symptoms, a common indication of early infection by *Xanthomonas* is the yellowing of leaves in a v-shaped lesion and the appearance of dark blackish veins [152]. Bacterial spot is an example of a disease caused by *X. cucurbitae* in cucurbits [151], *X. perforans* in tomato, and *X. gardneri* in peppers or tomatoes [150]. Foliar symptoms include small areas of discoloration. Fruits can become completely discolored and sunken if infected, ultimately reducing yield [151]. Untreated *Xanthomonas* infections eventually manifest in the mortification of entire plants, making them unharvestable. Black rot of hydroponic watercress is another well-documented result of infection by *X. nasturtii* [152]. The crop is grown almost exclusively via aquatic methods, but its short 15–20 day growth cycle, paired with consumption of the fresh foliage, limits possibilities for bactericides. Instances of the necrotic condition have been reported throughout North America and Europe [152].

Growers have several options to manage *Xanthomonas* infections in hydroponic systems. In the past, copper antimicrobial agents were a common management strategy for managing diseases caused by *Xanthomonas* [150]. However, as copper resistance has become increasingly prevalent and questions have arisen about its environmental impacts, research has shifted towards other solutions. Acibenzolar-s-methyl (ASM), streptomycin, and kasugamycin have shown positive effects in mitigating disease in tomato and pepper plants [150]. However, reports describe quick-developing resistance as a major problem. Information regarding the effectiveness of these methods in hydroponics systems is currently limited and would benefit from further research. As *Xanthomonas* is predominantly a seed-borne pathogen, a good preventative measure is testing seed lots and implementing proper sterilization of tools during plant maintenance [152]. Hawaii watercress producers have described disease management practices that effectively decreased disease in their hydroponic systems, including lowering humidity levels, increasing airflow, and reducing overhead irrigation [152].

### 3.2. Erwinia

As a genus of the *Enterobacteriaceae* order, *Erwinia* is a Gram-negative and non-sporulating bacteria. They are observed to produce small and distinct white colonies that grow best on yeast peptone dextrose adenine medium [190]. The pathogen primarily affects pears, apples, and other plants in the *Rosaceae* family. *E. pyrifolia* and *E. amylovora* were recognized from 1962 onward as considerable factors contributing to a decrease in strawberry production within the European Union [191]. From 1999 to 2014, strawberry production diminished by 6% regardless of the plot area remaining the same. Both of these strains cause a disease in plants called fire blight, after the scorched appearance they cause in plants. These species cause minor differences in the severity of symptoms [192], with *E. pyrifolia* being slightly less pathogenic. Fire blight was first reported in North America in 1780, although no causal agent was identified at the time, and has since been found in >40 countries [193]. Fire blight was originally described in both Turkey [193] and Korea [194] as a disease affecting only apple and pear trees and has now been noted in greenhouse-grown strawberry [190] and raspberry plants [194]. Multiple regions of the Netherlands experienced up to 40% crop loss in areas where symptoms appeared [191]. *Erwinia tracheiphila* is a non-soft rot-causing pathogen within the genus that causes wilt in cucurbits [153] and is transported by cucumber beetles [154]. The bacterial wilt of cucurbit crops has been noted as a major threat to production in the United States specifically [154].

*Erwinia* causes extensive crop damage to fruiting crops. The associated symptoms are black and brown discoloration in addition to the appearance of cankers that exude bacterial ooze [192]. These symptoms cause fruits to have a shiny or malformed appearance and lead to eventual necrosis. With very few differences in pathogenicity between the common strains *E. amylovora* and *E. pyrifolia,* molecular PCR protocols have been developed for their differentiation [192]. Upon further investigation using green fluorescent protein (GFP), the infection begins near natural plant openings or wounds before invading the xylem, parenchyma, and roots [195]. Similar to other plant pathogenic bacteria, *E. amylovora* relies on a type III secretion system. Exopolysaccharides, metalloproteases, and siderophores are additional virulence factors utilized by *Erwinia* [196]. Further research is required to fully expound all virulence strategies employed within this genus.

Disease management for *Erwinia* in hydroponic crops is a complex problem that needs to be solved. *E. amylovora* is known as a quarantine organism in many countries [197], whereas *E. pyrifoliae* is not, and therefore, the necessary screening for it is limited [190]. As with any pathogenic bacteria, good sanitary procedures and early detection are important disease management strategies for preventing an outbreak. There are many proposed biological and chemical control agents available, such as copper or the antibiotic streptomycin, but a limiting factor is the rapid development of antimicrobial resistance [197]. Several bacteria and fungi have been reported to provide protection from fire blight by outcompeting *Erwinia* for nutrients and space. These include *Pantonea agglomerans*, *Pseudomonas fluorescens*, *Bacillus subtilis*, and *Aureobasidium pullulans* [190]. Phage therapy has also emerged as a good alternative form of treatment for both *E. amylovora* and *E. pyrifoliae*. In Korea, an in vitro experiment was performed using the lytic bacteriophage phiEaP-8 [198]. The results demonstrated an ability to kill more than twenty strains of *Erwinia* paired with pathogen specificity and lack of impact on other related bacteria, including other non-pathogenic *Erwinia* species [198]. The results of this mitigation strategy in hydroponics systems require future examination but constitute an area of research worth investigating.

### 3.3. Agrobacterium

*Agrobacterium* is a genus of Gram-negative bacteria that is closely related to the plant growth-promoting bacterial taxa *Rhizobium*. *Agrobacterium* infections are not characterized primarily by their taxonomy but rather by plasmids that carry the pathogenic machinery. The pTi plasmid is the causative agent for crown gall disease in plants, which results in tumors forming at the plant crown [199]. As a horizontally transferred plasmid that spreads via bacterial conjugation, the pTi plasmid has also been observed in closely related genera such as *Rhizobium* [200,201]. The pTi plasmid is transferred to the plant via the VirB/VirD conjugation machinery and is integrated into the genome of the plant, where it begins to overproduce opines for its own metabolic benefit and produces phytohormones that trigger excessive plant cell proliferation [199]. The pRi plasmid, which is the causative agent of hairy root disease (also referred to as root matting), undergoes a similar DNA transfer process as the pTi plasmid [202]. The rolA-D genes of the pRi plasmid sensitize the plant to auxins and trigger excessive root growth, which can result in yield reductions of up to 10% in tomatoes [202].

While *Agrobacterium* is perhaps best known for causing crown gall disease, it is primarily hairy root disease that *Agrobacterium* causes in hydroponic greenhouses. Interestingly, it is biovar 1, the *Agrobacterium tumefaciens* species complex, that causes hairy root disease in hydroponic crops [155]. In soil, biovar 1 is typically the causative agent of crown gall, whereas biovar 2 is the group associated with hairy root disease [155]. Biovar 1 *Agrobacterium* has been found to cause hairy root disease in hydroponic tomatoes [137,138,155,156,157,158], cucumbers [155,157,158,159], and peppers [158]. The potential of rhizogenic *Agrobacterium* to cause large-scale hairy root disease is highlighted by the fact that 45% of Flemish greenhouses have biovar 1 *Agrobacterium* colonizing the roots of their tomato plants [203]. This does not suggest that 45% of the greenhouses have active hairy root disease because many lack the necessary pRi plasmid. However, these greenhouses act as a reservoir for disease to spread through if pRi is introduced. There are also reports of hairy root disease in lettuce; however, this is caused by *Agrobacterium* biovar 2 rather than the biovar 1 infections observed in fruiting crops [160]. An additional danger for hairy root disease induced by *Agrobacterium* is that it primes crops for secondary infections by other pathogens such as *Pythium* and *Pseudomonas* [157].

As a disease management strategy, fruit and vegetable greenhouse growers utilize hydrogen peroxide bubbled into the nutrient solution to control the growth of many pathogens. However, this strategy does not consistently work for *Agrobacterium* infections [204]. At 25 ppm, hydrogen peroxide is ineffective at controlling the population of *Agrobacterium* of any phenotype. Catalase-negative strains of *Agrobacterium* are sensitive to 50 ppm hydrogen peroxide, and catalase-positive strains are sensitive to 100 ppm. Given that 20–30 ppm is the standard concentration of hydrogen peroxide used to sanitize nutrient solution by growers, hydrogen peroxide is not a sensible option for controlling *Agrobacterium* [204]. Due to the ineffectiveness of hydrogen peroxide, biocontrol options for hydroponic *Agrobacterium* infections are actively being explored. Lytic phages present an opportunity to directly kill *Agrobacterium* in nutrient solution [205]. The OLIVR 1 phage decreased *Agrobacterium* abundance by four log units and demonstrated little evidence of resistance. For OLIVR variants where *Agrobacterium* can develop resistance, the resistance did not extend to other OLIVR variants. Inoculation of hydroponic greenhouse systems with *Paenibacillus xylanexedens* has also been proposed as a biocontrol option for managing hairy root disease caused by *Agrobacterium* [137,138]. The relative abundance of *Paenibacillus* was found to be negatively associated with the relative abundance of hairy root disease-causing *Agrobacterium* [165], and inoculation with *Paenibacillus* could suppress hairy root disease in greenhouse-grown tomato plants over two growing seasons^149^. Additionally, supplementation of calcium as calcium oxide at concentrations greater than 840 mg kg^−1^ can sensitize *Agrobacterium* to biocontrol agents [206]. While chemical agents may struggle to control hairy root disease and populations of *Agrobacterium*, biocontrol agents are demonstrating promise.

### 3.4. Ralstonia

*Ralstonia* is a recently established genus that was previously classified as *Pseudomonas* [161]. It is a Gram-negative bacterium that can cause many different types of disease in crops of both the necrotic and chlorotic nature [161]. The bacterium has a widespread geographical distribution but tends to be most prevalent in areas that practice monocropping and experience high humidity [161]. Unlike some other bacteria, *Ralstonia* is known to survive dormant in the soil and aquatic environments for long stretches of time, making it extremely difficult to eradicate [162]. Not only does this pathogen contribute to a severe loss of crop yield, ranging from 20 to 100% depending on the severity of the outbreak, but the long latency period has had indirect effects on fields and waterways [162].

Moko disease, brown and black rot, and bacterial wilt are three possible outcomes of infection by *Ralstonia solanacearum* [162]. The soil-borne pathogen [207] is a complex consisting of four phylotypes based on the plants they infect and the geographical location where they are found [162]. Pathogenicity relies on the type III secretion system as a primary mechanism of infection but also uses type II secretion systems [208]. It has been observed that of the reported strains, the number of type III effectors ranges from 45 to 76 [208]. Many types of effectors are used, including acetyltransferases, proteases, ligases, and effectors that interfere with ubiquitination processes, which work together to suppress plant defense strategies, evade recognition, and modify host metabolism [208]. Studies have been conducted to characterize the specific role of each effector in a variety of plant types. Most notably, this led to the observation of decreased disease progression in tomatoes and eggplants by downregulation of type III virulence effectors [209], which are both common hydroponically grown crops. Gene downregulation could have promising implications for disease management in situations where mainstream methods fail.

More broadly, *Ralstonia* enters at the root or wounds of the plant and travels through the xylem, where it can colonize and cut off the water supply [161]. This results in visible wilting and discoloration that leads to eventual necrosis [161]. The disease manifests somewhat differently, appearing as a darkening of vascular tissue [210]. Hydroponic potato production has been increasing annually, although the crop is typically grown below the optimal temperature range for *Ralstonia*. The host range of *Ralstonia solanacearum* is wide; it causes particularly severe economic damage in hydroponic tomato, pepper, cucumber, leafy greens, and potato production [161,162]. This presents a large problem in the hydroponics industry as the majority of these plants are favourably grown in soilless environments [189]. Worldwide, *Ralstonia* was estimated to account for the loss of USD 1 billion in potato sales [207].

There has been difficulty finding methods to effectively manage the infection and spread of *Ralstonia* in hydroponic systems. Numerous chemical agents have been tested, including pesticides, fumigants, bactericides [211], and metal-based nanoparticles [163]. While these compounds have generated positive preliminary results, the environmental impact, as well as quickly growing resistance, have indicated the need for research into more sustainable disease management practices [211]. Many newer methods can be characterized as biocontrol agents, including bacteria, fungi, and bacteriophages [212]. *Bacillus cereus* AR156 is one example of a beneficial bacteria that led to a 62.2% reduction in bacterial wilt in tomatoes caused by *Ralstonia solanacearum* [213]. *Trichoderma* spp. produce secondary metabolites found to inhibit *Ralstonia solanacearum* growth in vitro [214]. Possibly the most promising alternative has been phage therapy. Three waterborne lytic phages have been observed to work individually or together to reduce *Ralstonia solanacearum* in environmental water, as well as subdue many symptoms of bacterial wilt in planta [215]. Experimentation with Roma tomato plants showed a significant reduction in wilting percentages when they were inoculated with the phage. Specifically, plants irrigated with bacteriophage vRsoP-WF2 at a concentration of 10^8^ PFU mL^−1^ showed no disease symptoms compared to the control, which showed wilting in over 40% of plants [215].

### 3.5. Clavibacter

Species that are categorized within the Gram-positive genus *Clavibacter* are among the most devastating plant pathogens due to a lack of adequate bactericides to eliminate them [165]. At least two species, including *C. michiganensis* and *C. sepedonicus*, are listed as quarantine organisms and require special directives by the European Union to both detect and handle infected plants [216]. With a latency period of up to 40 days [216] and unreliable identification methods, the only promising control measures currently consist of crop rotation and good sanitation procedures [217].

The seed-borne pathogen [166] is the cause of bacterial canker, one of the most significant diseases of hydroponic tomato crops in the United States and Canada [217]. The pathogen spreads through roots within hydroponics systems, especially when using NFT [217]. Unlike other phytopathogens, *C. michiganesis* can infiltrate crops without openings or wounds and infect entire greenhouses [218]. The bacterium is also known to infect peppers, corn, wheat, and potatoes [166]. Symptoms of the disease range from wilting and cankers to dark rings and bacterial ooze, depending on the host plant [219]. The mechanism of infection for *Clavibacter* is unique compared to most phytopathogens. Based on current research, the bacteria lack type III secretion systems to transfer proteins directly into host cells [220]. The use of GFP revealed that separate genes and virulence factors contribute to blister formation and wilting symptoms [220]. A multitude of cell-wall degrading enzymes and serine proteases are thought to be at the core of disease [220].

Traditional chemical agents for pathogenic bacteria have proven unsuccessful in mitigating *Clavibacter* symptoms, and there are currently no resistant cultivars available [218]. Management of the canker-forming disease is under the authority of growers, who have to rely on seed testing and crop rotation [217]. However, there have been reports of a simple and effective method that takes advantage of the bacterium’s low tolerance for acidic pH levels. A study involving over 100 tomato plants grown in an NFT hydroponics system analyzed the effects of lowering nutrient solution pH as well as soaking seeds in acidic solutions before use. To adjust the pH, monosodium phosphate was used [217]. The results showed that at a pH of 6.5, 34 of 48 plants still developed canker symptoms, whereas when the pH was lowered to 5.0, only 11 of 48 plants showed signs of disease [217]. In hydroponics, the pH of the nutrient solution can be readily manipulated, making it a promising disease management option for plants being grown in commercial hydroponic greenhouses.

### 3.6. Pectobacterium

*Pectobacterium* is a genus of Gram-negative necrotrophic plant pathogens that were formerly classified as members of the genus *Erwinia* [221]. Due to high levels of genetic variation in the genus, detection and classification of *Pectobacterium*—particularly with the use of nucleotide primers—is difficult [222]. Soft-rot pathogenesis caused by *Pectobacterium* is mediated by plant cell wall-degrading enzymes [223], which include cellulases, hemicellulases, pectinases, and proteinases. These enzymes are secreted onto plant surfaces through type II secretion systems [224]. In contrast to phytopathogens that rely on host-specific type III secretion systems to be infective [149], necrotrophic phytopathogens tend to have broader host ranges [223]. Rather than being limited by host range, *Pectobacterium* infection requires a critical bacterial population density threshold, or it will prematurely activate plant immune responses [225]. *Pectobacterium* utilizes quorum sensing to activate the production and secretion of plant cell wall-degrading enzymes [226], ensuring that the bacterial population density is sufficient to trigger soft rot disease.

Multiple species of the genus *Pectobacterium* are known to cause disease in crops commonly grown in hydroponic greenhouses. Hydroponic watering systems allow for the rapid spread of *Pectobacterium* from infected plants to healthy ones due to shared water reservoirs and recirculating nutrient solutions [222]. Leafy green vegetables are a major target of *Pectobacterium* infections, with soft rot being found in hydroponic lettuce [172,173,174], cabbage [175,176], and kale [177]. *Pectobacterium* also causes soft rot in many of the major hydroponically grown fruiting crops, including tomato [168,169], cucumber [170,171], pepper [171], and eggplant [227]. Instances of disease were primarily caused by *P. carotovorum*. However, *P. brasiliense* [167,168,169,170,177] and *P. wasabiae* [227] can also cause disease in greenhouse-grown crops.

A number of disease management strategies have been tested due to the devastation that *Pectobacterium* can inflict on hydroponically grown fruit and vegetable crops. One such strategy is the use of carbon dioxide microbubbles in the nutrient solution to inactivate *Pectobacterium carotovorum* subsp. *carotovorum* infection of crisphead lettuce [172]. Similarly, microbubbling of ozone can also inactivate *P. carotovorum* subsp. *carotovorum* in hydroponic nutrient solution [228]. The aforementioned strategies for disease management rely on directly suppressing the growth of *Pectobacterium*, but it also may be possible to improve the resistance of plants to *Pectobacterium* infection. Additional supplementation of soluble calcium to hydroponically grown cabbage improves resistance to soft root rot caused by *P. carotovorum* subsp. *carotovorum* and extend protection to post-harvest spoilage [176]. Biocontrol agents have also shown promise for managing *Pectobacterium* infections. The protist *Nitrosocosmicus oleophilus* can induce systemic resistance in *Arabidopsis thaliana*, which confers resistance to *P. carotovorum* [229]. Immune responses against *Pectobacterium* can be triggered by the application of homoserine lactones [230] or sulfur nanomaterials [174], which trigger the salicylic and jasmonic acid pathways in plants and activate defensin genes that can sequester iron away from *P. carotovorum* subsp. *carotovorum* [231]. Defensins can also be exogenously applied in the form of powdered immunized insects (*Protaetia brevitarsis seulensis*, *Hermetia illucens*, and *Gryllus bimaculatus*), which are rich in defensins and can result in 10% yield increases when applied in lettuce as a result of disease mitigation [173]. Combinations of strategies should be employed in instances where soft rot is detected in hydroponic greenhouses to keep *Pectobacterium* populations below the population density where soft rot disease is triggered and to ensure that the immune systems of plants are primed to resist disease symptoms.

### 3.7. Pseudomonas (Phytopathogenic)

**Figure 2:**
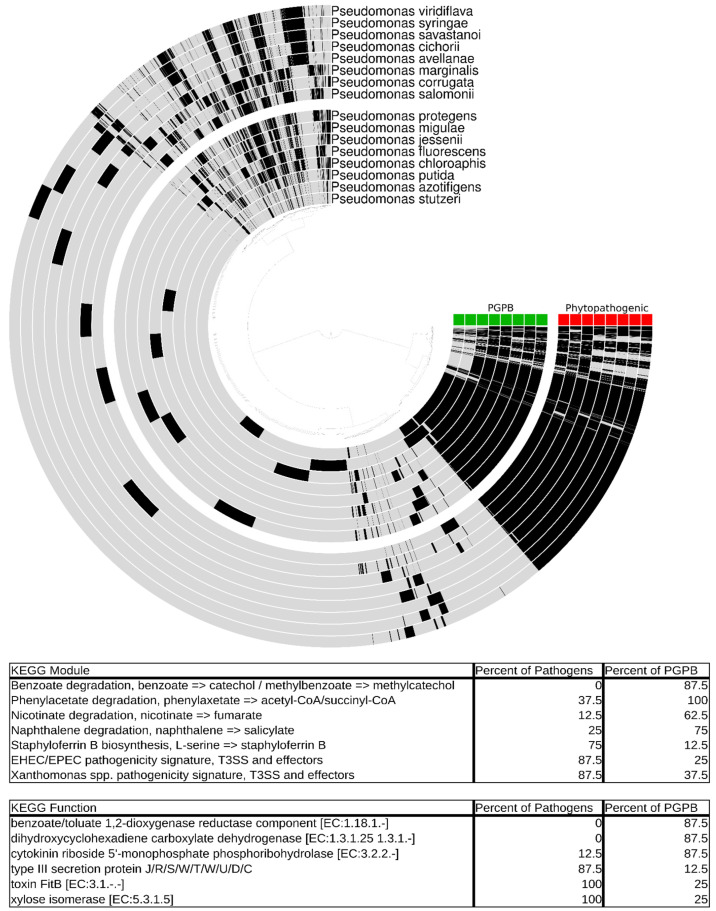
A circular phylogram of the pangenome of sixteen *Pseudomonas* reference strains, eight of which are considered plant growth-promoting (*P. protegens, P. migulae, P. jessenii, P. fluorescens, P. chloroaphis, P. putida, P. azotifigens*, and *P. stutzeri*) and eight that are considered phytopathogenic (*P. viridiflava, P. syringae, P. savastanoi, P. cichorii, P. avellanae, P. marginalis, P. corrugata*, and *P. salomonii*). The black shading of a cell represents the presence of a gene cluster in the genome of the species. Tables below the phylogram highlight KEGG modules and genes of interest that are enriched in either the PGPB or phytopathogenic species of *Pseudomonas*.

While many *Pseudomonas* species have been noted for their ability to promote plant growth, there are also many phytopathogenic species that belong to the genus. Mirroring the diversity of the genus, *Pseudomonas* phytopathogens have a diverse set of mechanisms to trigger disease in plants [232]. As displayed in a pangenomic analysis of a subset of PGPB and pathogenic *Pseudomonas* species (Figure 1), the differences in gene content often do not follow pathogenicity but rather taxonomy. This is particularly evident in the *Pseudomonas fluorescens* subgroup (*P. marginalis*, *P. corrugata*, *P. salomonii*, P. *protegens*, *P. migulae*, *P. fluorescens*, *P. chlororaphis*), where the pathogens have gene clusters patterns that more resemble the PGPB of the same group than the pathogens of the *Pseudomonas syringae* subgroup (*P. viridiflava*, *P. syringae*, *P. savastanoi*, *P. cichorii*, and *P. avellanae*). The primary mediator of *Pseudomonas* infections is the type III secretory pathway, which delivers effectors and modulates the immune system of plants to promote infection [143]. When scrutinizing the presence–absence of gene clusters between PGPB and pathogenic *Pseudomonas* species, type III secretion systems were indeed the most apparent gene signature of pathogenicity. While plant growth-promoting species of *Pseudomonas* mostly lack type III secretion systems, the overwhelming majority of phytopathogenic *Pseudomonas* have secretion systems encoded in their genomes (Figure 2). Other enriched genes in phytopathogenic species include toxin–antitoxin and chemotaxis genes, which are critical for mediating pathogenesis [233]. In contrast, many of the genes enriched in PGP *Pseudomonas* species are those related to aromatic hydrocarbon detoxification and plant hormone activation (Figure 2) [234]. Phytopathogenic *Pseudomonas* strains also produce a wide variety of phytotoxins that can contribute to a broad spectrum of symptoms [235]. For example, phaseolotoxin, produced by *P. syringae* pv. *phaseolicola* inhibits a critical step of the urea cycle and causes the buildup of ornithine and a deficiency of arginine in plant cells, which results in chlorosis [141]. Another example of a *Pseudomonas* phytotoxin is syringomycin, which forms a pore in the cell membrane of plants and induces tissue necrosis [236]. Like *Agrobacterium*, phytopathogenic *Pseudomonas* modulates auxin production to alter plant physiology in ways that are beneficial to it [237]. Similar to another pathogenic clade—*Pectobacterium*—some strains of *Pseudomonas* utilize pectinases to induce soft rot of plants [238]. The shared pathways and phenotypes that phytopathogenic *Pseudomonas* strains induce in plants make differentiation from other pathogens difficult, complicating diagnosis.

A number of *Pseudomonas* species have been found to cause disease in core hydroponic greenhouse crops. *P. capsici* causes leaf spotting and blight in tomato, pepper, eggplant, cabbage, and lettuce in greenhouse conditions [179]. Many strains of *P. capsici* display resistance to copper treatment, which complicates disease management strategies [179]. *P. cichorii* is the causal agent of midrib rot in greenhouse-grown lettuce [184]. Two phylogenetically similar species, *P. corrugata* and *P. mediterranea,* induce pith necrosis in greenhouse tomato and pepper plants using similar biological pathways [19,180,181]. The *P. syringae* species complex is the causative agent of bacterial speck in tomatoes [182], bacterial leaf spot in kale [185], and angular leaf spot in cucumber [183].

Disease management strategies exist to minimize the spread of phytopathogenic *Pseudomonas* in greenhouses. Many pathogenic *Pseudomonas* species are transferred through infected seeds, which, in theory, makes pathogen-free seeds the most effective management strategy [239]. However, in practice, this is nearly impossible to achieve; thus, disease surveillance and treatment are necessary practices. A number of PCR-based detection methods for *P. syringae* have been developed but suffer from poor limits of detection [239]. A similar method, known as loop-mediated isothermal amplification, can be completed without extracting genomic DNA and is therefore more easily performed with environmental or low biomass samples suspected to contain *P. syringae* [183]. Management strategies have been complicated for phytopathogenic *Pseudomonas* because many strains display resistance to copper treatments [179,240]. Consequently, direct antibacterial activity or stimulation of the innate immune system of plants has been explored with compounds such as acibenzolar-S-methyl [240], para-aminobenzoic acid [241], and zinc oxide nanostructures [242]. Additionally, modification of the lighting spectrum using polythene glazing has been shown to mitigate bacterial speck in greenhouse-grown tomatoes by 10% [243]. Biocontrol once again shows promise as a means of phytopathogen management, with multiple species of *Bacillus* displaying antagonistic effects against *P. syringae* [244]. Effective management strategies will ultimately be influenced by the disease observed and the strain of *Pseudomonas* causing the disease, given the vast genetic diversity of phytopathogens that belong to the genus.

## 4. Conclusion and Future Directions

The bacterial microbiome within hydroponics systems is an important area of study for maximizing crop yields and mitigating damage from phytopathogens. Beneficial and phytopathogenic bacteria relevant to the hydroponic sector can exist within the same taxonomic genus. Acquisition of a single plasmid by *Agrobacterium* strains can be the difference between benign and disease-causing outcomes, highlighting the complexity of the plant microbiome and the need for further studies. A pattern detected during the research for this review included the over-reliance on in vitro studies combined with the underuse of to-scale plant trials. Although testing the biochemical capabilities of bacteria in the lab is critical, the results do not directly translate to a commercial hydroponic environment. Microbes may display certain characteristics in a petri dish but exhibit contradicting ones in a greenhouse. Thus, more research is required to determine the effects of bacterial inoculants in the relevant environments. Further, many studies used small numbers of plants or did not allow the plants to grow through their crop cycle to maturity, thus failing to measure the impact of the experiment on yield. Where possible, we would encourage larger-scale plant trials that more closely mirror commercial hydroponic farming. In the commercial agricultural sector, it is irrelevant to a grower if a PGPB significantly increases the size of a strawberry plant’s leaves if it does not also improve the size, yield, or quality of the berries. One cannot conclude that a bacterium is a PGPB of commercial value if it does not increase the yield of the marketable product.

Phytopathogen research is also plagued by a dearth of primary studies of the mechanisms of disease. Much of the literature is built upon observations of disease in commercial greenhouses. The isolation of the pathogens for further study is critical for understanding their pathology in plants. Running inoculation trials in crops is a costly but necessary process to understand the pathogen density required for disease, the breadth of crops they can affect, and effective disease management strategies, which also needs to become a research focus of its own. Many current management strategies are becoming obsolete as phytopathogens develop resistance and regulations limit the application of antimicrobial compounds. As such, further research and development of biocontrol agents is a promising avenue to sidestep these limitations. However, commercialization of biocontrol agents is also limited by long and difficult regulatory requirements. Action, both by researchers and legislators, must be taken to ensure that hydroponic fruit and vegetable growers have access to the effective and safe tools they need to prevent crop loss by the pathogens mentioned in this review.

Hydroponics system usage has been increasing annually; thus, research in this area will have an increasing impact on contributing to global food production systems. Researchers are encouraged to consider hydroponic plant microbiomes as an incredibly diverse and interesting field of study. Going forward, research should focus on microbial consortia, as microbial interactions can offer additive or synergistic effects if designed successfully, further improving crop yields [26]. In essence, the hydroponic industry is of critical importance in feeding the growing world population. Research in this sector has the potential to yield high-impact results. The hydroponic bacterial microbiome is a complex research area that would benefit from additional scrutiny. Bacteria are both invisible friends and foes within the global food production system. Developing elegant solutions to increase crop yields via the introduction of beneficials and the limitation of phytopathogens holds immense potential to shape the sustainable future of the agriculture industry.

## Figures and Tables

**Table 1:** Summary of plant growth promotion mechanisms exhibited by reviewed plant growth-promoting bacterial genera that are relevant to hydroponics systems.

Genus	ACC Deaminase	Nitrogen Fixation	Siderophores	Phyto- Hormone Production	Biocontrol	Nutrient Solubilization
*Pseudomonas*	✓ [32]	✓ [32]	✓ [32,33]	✓ [34]	✓ [35,36,37,38]	✓ [32]
*Bacillus*	✓ [39,40]	✓ [41]	✓ [42,43]	✓ [43,44]	✓ [45]	✓ [43]
*Azospirillum*	✓ [46]	✓ [47]	✓ [48]	✓ [49]	✓ [50]	✓ [51,52]
*Azotobacter*		✓ [53]	✓ [53]	✓ [54]	✓ [55]	✓ [54]
*Rhizobium*		✓ [56]	✓ [57]	✓ [58]	✓ [57]	✓ [58]
*Paenibacillus*		✓ [59]	✓ [60]	✓ [59]	✓ [61]	✓ [61]
*Paraburkholderia*	✓ [62]	✓ [63]	✓ [63]	✓ [64]	✓ [63]	✓ [63]

**Table 2:** Summary of seven genera of phytopathogenic bacteria that cause disease in four major hydroponic crops. Checkmarks denote which crop types bacteria cause known economically important disease symptoms.

Genus	Tomato	Pepper	Cucumber	Leafy Vegetables
*Xanthomonas*	✓ [150]	✓ [150]	✓ [151]	✓ [150,152]
*Erwinia*			✓ [153,154]	
*Agrobacterium*	✓ [137,138,155,156,157]	✓ [158]	✓ [155,157,159]	✓ [160]
*Ralstonia*	✓ [161,162]	✓ [161,162]	✓ [163]	✓ [164]
*Clavibacter*	✓ [165]	✓ [166]		
*Pectobacterium*	✓ [167,168]	✓ [169]	✓ [170,171]	✓ [172,173,174,175,176,177]
*Pseudomonas*	✓ [178,179,180,181,182]	✓ [178,179,180]	✓ [183]	✓ [184,185]

## Data Availability

*Pseudomonas* pangenome was computed using the following NCBI RefSeq genome accessions: GCF_000452845.1, GCF_000517305.1, GCF_001708425.1, GCF_008692035.1, GCF_013386885.1, GCF_020917325.1, GCF_018394375.1, GCF_900184295.1, GCF_000425625.1, GCF_014524625.1, GCF_900215245.1, GCF_900104905.1, GCF_900106025.1, GCF_900560965.1, GCF_000412675.1, and GCF_019704535.1. Construction of the pangenome was performed using anvi’o following the pangenomics workflow: https://merenlab.org/2016/11/08/pangenomics-v2/ (accessed on 30 July 2024).
